# Phalloplasty in Children with Severe Penile Tissue Loss: Single Center Case Series

**DOI:** 10.3390/medicina61071124

**Published:** 2025-06-22

**Authors:** Gokhan Demirtas, Suleyman Tagcı, Derya Yayla, Hasan Murat Ergani, Gunay Ekberli, Bilge Karabulut, Huseyin Tugrul Tiryaki

**Affiliations:** 1 Pediatric Urology Department, Ministry of Health, Sincan Government Hospital, 06930 Ankara, Turkey; drgokhandemirtas@gmail.com; 2 Pediatric Urology Department, Ankara Bilkent City Hospital, University of Health Science, 06800 Ankara, Turkey; suleyman_tagci@hotmail.com (S.T.); bilgekarabulut@hotmail.com (B.K.); httiryaki@hotmail.com (H.T.T.); 3 Pediatric Urology Department, Gaziantep City Hospital, 27470 Gaziantep, Turkey; dryayla@yahoo.com; 4 Plastic and Reconstructive Surgery Department, Ankara Bilkent City Hospital, University of Health Science, 06800 Ankara, Turkey; dr.hasanmrt_06@hotmail.com

**Keywords:** agenesis of the penis, amputations, traumatic, reconstructive surgical procedure, children

## Abstract

*Background and Objectives:* Penile tissue loss, which can be an acquired condition due to trauma or infection, but is also seen in congenital anomalies, is a rare condition in children. A standard surgical approach is often not possible due to the different degrees and etiologies of penile tissue loss. The continuing growth and the presence of various congenital anomalies in children require a different penile reconstruction approach than in adults. We aimed to share our experience and surgical results with children in whom we performed penile reconstruction with different techniques due to penile tissue loss. *Materials and Methods:* Ten cases that underwent penile reconstruction between 2018 and 2023 were evaluated retrospectively. Age at initial operation, associated anomalies, surgical technique, and other related surgical attempts, as well as functional and cosmetic results, were recorded. *Results:* Ten boys aged between 6 months and 17 years underwent phalloplasty due to penile tissue absence. In six cases, penile tissue loss was due to acquired causes, and in four cases, congenital anomalies were the reason. The most common cause of penile tissue loss was circumcision complications. In four cases, penile reconstruction was achieved by mobilization of the remaining corpus cavernosum tissues, in two cases, the cavernous tissue was adequate and repaired with glansplasty and penile skin graft. Phalloplasty was performed by tubularization of a skin and subcutaneous fat flap, removed from the pubic region and scrotal region, in two cases. A microvascular radial forearm flap was performed in a 17-year-old patient with penile tissue loss because of trauma, and a free skin flap taken from the forearm was used for penile reconstruction. Thirty percent of patients required a second surgery. Urinary continence was present in eight of the cases. Although four cases were classified as cosmetically unsatisfactory in our evaluation, all patients and their families reported being satisfied with the cosmetic results. *Conclusions:* Penile reconstruction for penile tissue loss in children should be performed in clinics where different scenarios can be applied. With maximum preservation and mobilization of existing cavernous tissues, temporary penile reconstruction with local flaps should be performed in young children at an early stage to minimize the psychological effects of penile absence. Although an esthetically perfect result cannot be guaranteed, patients and families are generally satisfied with the outcome.

## 1. Introduction

Penile tissue loss in children is a rare condition that occurs in varying degrees [1,2,3]. Since it is not possible to create a functional penis structure in the event of penis loss, male children born with aphallia, or who later lose their penises, have even been subjected to feminizing genitoplasty operations to convert them to the female gender [1,2,3,4]. However, today it is no longer considered acceptable to genetically and sexually transform a male child into a female child. Phalloplasty for penile insufficiency in biological males aims to provide a functional phallus by incorporating as much natural penile tissue as possible.

In cases of aphallia, rudimentary penis/micropenis, trauma, iatrogenic acquired aphallia, or cloacal exstrophy in children, phalloplasty is attempted to create a functional and esthetically pleasing penis [5,6,7,8,9]. While phalloplasty using microvascular radial forearm transfer flaps is preferred in adult patients [8], it can only be applied to post pubertal patients in multidisciplinary clinics in children. However, temporary alternative solutions are sought for younger children. In particular, local skin flaps are used in phalloplasty procedures to improve the psychological well-being of children and their families, especially in cases where there is no penis [6,7]. In children who will grow and develop, preserving the existing cavernous tissues as much as possible can provide sufficient penis length in adulthood. These patient-specific solutions should be performed by adhering to the general basic principles of phalloplasty (such as preserving the existing cavernous tissue as much as possible).

There are few studies in the current literature on pediatric penile reconstruction due to penile tissue loss [1,2,3,4,5,6,7]. Since there are not enough publications in the literature to guide penile reconstructions in children due to penile tissue loss, we retrospectively reviewed phalloplasty surgeries performed with different approaches in pediatric cases with different degrees of congenital or acquired penile tissue loss in our clinic in the last six years. The presented study aims to share our experience in pediatric phalloplasty, as well as functional and cosmetic results.

## 2. Materials and Methods

This study was performed in line with the principles of the Declaration of Helsinki. The study was approved by the Ethics Committee of Ankara Bilkent City Hospital (Approval date: 12 February 2025, Approval code: 1-25-1003). Informed consent forms were obtained from all patients and caregivers. Children who underwent penile reconstruction due to congenital or acquired penile tissue loss between 2018 and 2023 were retrospectively evaluated from hospital records. The inclusion criteria for our study were patients under the age of 18 who underwent penile reconstruction due to penile tissue loss. The patients’ ages at the time of phalloplasty, the cause of penile tissue deficiency, phalloplasty technique, and the type of graft or flap used were recorded. An attempt was made to determine how a patient-specific approach should be planned for penile tissue loss detected for various reasons and in different severities. Complications detected during perioperative and follow-up were noted. Patients were asked whether they urinated spontaneously from the urethra and whether they were continent. The appearance of the penis obtained after the operations was evaluated (excellent, good, adequate, poor). It should be noted that no standardized scale was used in the evaluation of cosmetic results. Cosmetic evaluation was performed as follows: acceptable penile appearance—excellent; presence of near-normal penile shaft and glans—good; deformed glans, short but adequate penile shaft—adequate; no glans, short penile shaft—poor. Additionally, all patients and their families were asked whether they were satisfied with the esthetic and functional results.

## 3. Results

Surgical interventions were planned specifically for the patient, taking into account age, clinical condition, etiology, severity of penile tissue loss, presence/absence of cavernous tissue, presence/absence of urethral involvement, and presence/absence of the glans penis.

Patients with a total absence of cavernous tissue required penile replacement. In the presence of cavernous tissue, different surgical techniques that preserve the existing tissue as much as possible were preferred.

**Figure 1:**
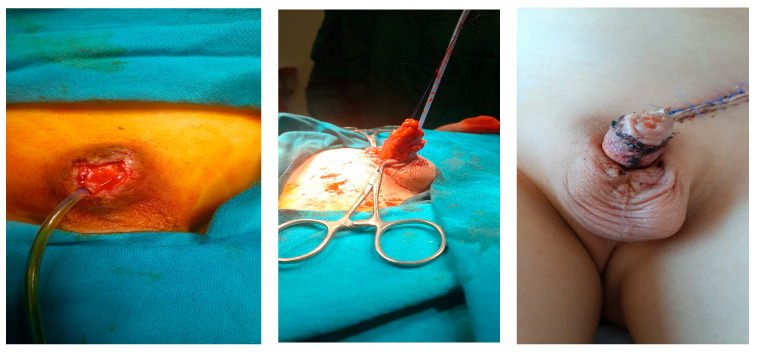
Circumcision complication.

**Figure 2:**
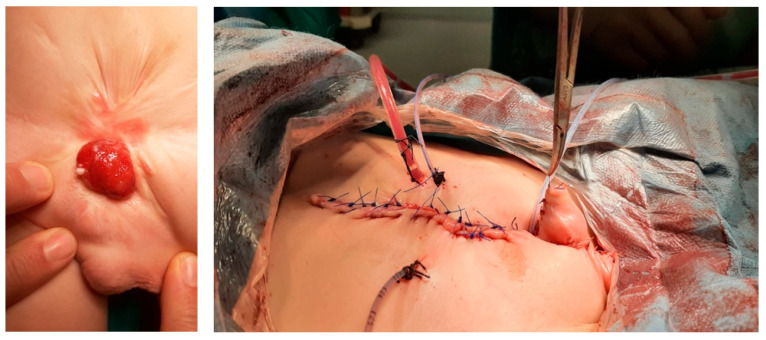
Exstrophy vesica +aphallia case.

**Figure 3:**
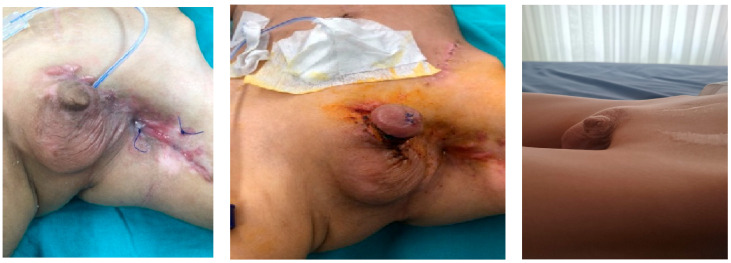
Gunshot case.

**Figure 4:**
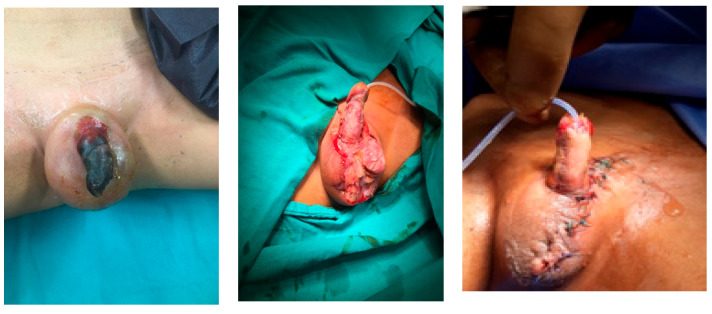
Fournier’s gangrene case.

**Figure 5:**
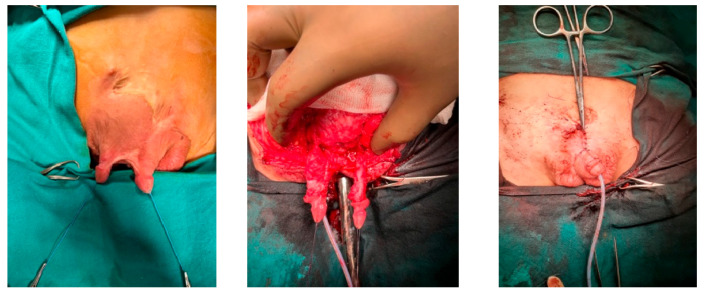
Extrophy vesica and diphallus case.

**Figure 6:**
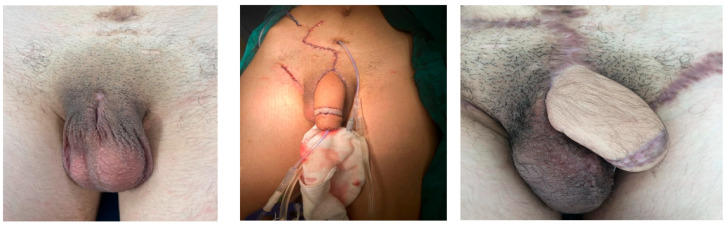
Microsurgical forearm graft phalloplasty.

Phalloplasty was performed on 10 boys aged between 6 months and 17 years due to penile tissue failure (Table 1). Penile tissue loss was determined to be due to acquired causes in six cases and congenital anomaly in four cases. Etiologic factors were as follows: complication of circumcision involving monopolar cautery (n = 2) (Figure 1), rudimentary penis (n = 1), hypospadias surgery (n = 1), exstrophy vesica combined with aphallia (n = 1) (Figure 2), gunshot injury (n = 1) (Figure 3), tissue loss due to Fournier’s gangrene (n = 1) (Burkitt lymphoma) (Figure 4), extrophy vesica and diphallus (n = 1) (Figure 5), extrophy cloaca (n = 2), complete loss of the penis due to trauma (n = 1) (Figure 6).

In our series, patients have been followed up for 2–7 years, with an average of 4.5 years. In four cases, our approach involved preserving the existing cavernous tissues using local skin flaps or grafts in order to achieve penile reconstruction. In all four cases, penile enlargement was achieved by mobilization and advancement of the remaining corpus cavernosum. This was accomplished by circumferential dissection of the corpus from the surrounding wound and by division of the suspensory ligament of the penis in two cases, with the remaining corpus cavernosa completely separated from the pubic bones in three cases. In two cases, repair was completed with glanuloplasty and penile skin grafting because the cavernous bodies were adequate.

In the diphallus case, the penile tissue in which there was no continence was excised. The cavernous bodies of the penis in which there was continence with the more normal urethral pathway and standard bladder configuration were dissected. The operation was completed by pulling them to the midline and covering them with a local skin graft.

Penile replacement was performed in three cases. In the two cases with a congenital absence of cavernous tissue, phalloplasty was performed by tubularization of a flap of skin and subcutaneous fat elevated from the pubic region and scrotal region. Microvascular radial forearm flap was carried out in the case of a 17-year-old patient with penile tissue loss resulting from trauma, and a free skin flap harvested from the forearm was used for the reconstruction of the penis (Table 1).

Penile ischemia was detected in four cases at the time of admission, and in addition to debridement, local dressing, and care, 20 sessions of hyperbaric oxygen therapy were administered to try to save maximum cavernous tissue. The remaining cavernous tissue was found to be sufficient in three cases and insufficient in one case despite all attempts. Three cases required additional surgical intervention.

While seven of the cases had urinary continence, the case with cloaca urinated through the urethra but was incontinent; the case with bladder exstrophy had a closed bladder neck, and urinary drainage was achieved via appendicovesicostomy; and the case with a forearm free skin flap, a vesicostomy was created to achieve urine drainage. Penile appearance was evaluated in four different categories (excellent, good, adequate, poor). In four cases, the penile appearance was assessed as poor, in two cases as adequate, and three cases as good. One case was classified as excellent (Table 1). All patients and their families reported to be satisfied with both the esthetic and functional results.

## 4. Discussion

Penile tissue loss in children is rare and occurs in very different degrees. In addition to congenital anomalies, genital trauma is the most crucial cause of penile tissue loss in the pediatric age group [10]. Casey et al. reported in their study that children between the ages of 5 and 8 years are more prone to genital trauma (37.1%) [11]. Previously, young children who lost their phallus completely through amputation were converted to the female gender [1,4]. Today, the conversion of a biologically male child to the female gender due to loss of penile tissue is not an acceptable option. However, since penile tissue loss occurs to different degrees, there is no universal treatment strategy. Treatment typically requires standard urological care combined with the surgeon’s creativity and innovation. The goals of penile reconstruction in the pediatric age group include obtaining/preserving sufficient erectile tissue, creating a urethra that reaches the tip of the penis, and covering the penis with skin by glans recreation [12,13,14,15]. Achieving the goals mentioned above through surgery provides the patient with a cosmetically acceptable penis and the ability to urinate normally. In addition, improving the child’s body image and reducing family anxiety are essential reasons for early surgical decision-making [16]. In cases of complete penile tissue loss or congenital absence, our preference is to perform temporary phalloplasty before puberty. Collaboration with the family is vital because the condition may require long-term follow-up and treatment. We believe that providing detailed information to caregivers before surgery will facilitate the management of cosmetic and functional problems that may develop after surgery.

The penis has several unique anatomic features that must be considered during reconstruction. A hairless, elastic appearance of the penile skin that can accommodate growth is essential for proper erection [17]. Superficial wounds can be treated initially with wound dressings or sutures after exploration. In the management of severe penile skin defects, the shaft of the penis is covered with free skin grafts obtained from hairless areas. There are case reports in the literature in which hair growth in the urethra or penile shaft has been observed after long-term follow-up, and laser ablation has been recommended [18,19]. In our series, the penis shaft was covered with skin graft in six cases. During follow-up, we observed that all skin grafts were adherent and had sufficient elasticity. More extensive injuries require evaluation of both the urethra and the corpus cavernosum. Given the long life expectancy of the patient group, repair of the cavernous structures and the urethra requires precision, and the repair technique must be determined appropriately. As a standard approach to penile tissue loss, all penile tissues are preserved as much as possible, with the maximum benefit mainly derived from the cavernous tissue during reconstruction. The use of the corpora cavernosa stump is recommended as a first-line treatment [1]. In cases of partial cavernous body loss, additional length is obtained by dissecting the cavernous bodies from the pubic bone and suspensory ligament release [20,21]. In four of our cases, an adequate penile length was attempted to be achieved by cavernous body dissection. In three cases, except for the case with cloacal exstrophy, a penile length of 4–5 cm was achieved. In two cases, repair was performed by glans reconstruction.

Reconstructive surgery consisting of a tubularized abdominal flap with autologous rib fascia for penile injuries was first described by Bogoraz in 1936 [22]. Various flap techniques described in the literature are generally accepted as the gold standard for adult patient groups requiring phalloplasty for different reasons [23,24,25]. Most of these techniques require a micro-surgical approach with a long operative time and multiple complications. Implementation of these techniques in children is challenging. Temporary non-microsurgical penile reconstruction with local or free transfer flaps or other grafts has been described for patients who have suffered a penile injury for any reason during the growth and development period [26,27,28]. In our series, reconstruction using the remaining erectile tissue was almost always possible in the majority of cases. If the cavernous bodies cannot be preserved despite all protective and salvage efforts or are congenitally absent, our preferred method is reconstruction using a free flap taken from the forearm in adolescent cases. At the same time, we prefer temporary reconstructions with a pubic or scrotal local skin flap in young children.

De Castro et al. performed phalloplasty with the abdominal flap technique in infants younger than 2 years of age. This technique was reported by Bettocchi et al. for patients with gender dysphoria and included urethroplasty. De Castro et al. suggest abdominal flap phalloplasty alone (without urethroplasty) to be simple and without complications [28]. The factor that limits the feasibility of the technique is the presence of an anterior abdominal wall defect accompanying congenital anomalies that will require penile reconstruction, like exstrophy vesica and persistent cloaca. Considering its necessity, the anterior abdominal wall should be handled carefully; otherwise, alternative options should be sought. In the presented study, only one 17-year-old patient underwent a free radial forearm flap with urethroplasty. He had a cosmetically and functionally acceptable penis until urethral stricture on his postoperative first year.

Oliveira et al. reported results of surgical neophalloplasty using scrotal flaps for aphallic children. As a result, they claim this technique to be reproducible and straightforward for creating a temporary neophallus in prepubertal boys [27]. Cezarino et al. recently reported their multi-institutional experience and long-term follow-up of patients with penile agenesis treated with scrotal flap phalloplasty. According to their experience, scrotal flap phalloplasty is a simple, minimally invasive, temporary reconstruction in the pediatric population [16]. Scrotal flap phalloplasty was performed on a patient with aphallia and suprapubic skin flap phalloplasty was performed on a patient with a rudimentary penis in the presented study. These two patients are now in the prepubertal period. When these two patients reach adolescence, they will be evaluated to see if they need phalloplasty or if a penile implant can be placed on the existing phallus.

On the remaining seven patients, penile reconstruction was completed with local skin grafts, cavernous body dissection, and glans reconstruction techniques alone or in combination, depending on the degree of tissue loss.

In our series, urethroplasty was performed simultaneously with phalloplasty in one patient. Unfortunately, although he was continent, the long-term outcome was a urethral stricture, resulting in the creation of a vesicostomy. Cezarino et al., in their series consisting of patients with penile agenesis, preferred to perform urethroplasty in a definitive operation [16]. Oliveire et al. and De Castro et al., in their series, also do not recommend performing urethroplasty simultaneously with phalloplasty [27,28].

In the remaining patients, the existing urethra was dissected and attempted to be transferred to the tip of the penis. In one patient, the need for urethral dilatation was determined during follow-up. All patients were continent except for those with exstrophy vesica and cloaca. In the case with bladder exstrophy, the bladder neck closed, and urinary drainage was achieved from appendicovesicostomy. The patient with a forearm free skin flap because of urethral stricture, urinary drainage was achieved with vesicostomy.

In our evaluation, unfortunately, four of our patients had poor penis appearance, but six patients had an adequate and good penis appearance. We did not detect a completely normal image in any patient. All patients and their families stated that they were satisfied with the esthetic. Although our penis cosmetic evaluations are not good in our opinion, patients and families have expressed that they are pleased with the appearance of the penises they have achieved after the initial horrifying images.

The main limitations of the study are small sample size, retrospective design, and lack of long-term results due to the age of the patient population.

## 5. Conclusions

According to our experience, it can be claimed that in the repair plan, care should be taken to preserve penile tissues as much as possible and use them in reconstruction. For infants and young children, local flaps/grafts used in phalloplasties and proper urine evacuation techniques are suitable solutions that reduce the anxiety of the child/family until the appropriate age. However, the standard procedures described are often not possible to perform, and achieving a satisfactory outcome can be difficult in many patients due to pathologies accompanying penile tissue loss (such as exstrophy cloaca or vesica). Unique anatomic and physiologic structure of the penis, the etiology of penile tissue loss, and even patients’ age makes operative techniques ‘’patient specific’’. Most patients need complex, modified, and sometimes combined techniques that require experienced centers.

Considering the long-life period of the managed population, the remaining tissue must be handled carefully for future definitive reconstructions, and caregivers should be informed in detail regarding future expectations.

## Figures and Tables

**Table 1:** Treatment according to causes and type of penile injury.

Age	Cause	Type of Tissue Loss	Treatment		Continence	Cosmetic Appearance	Secondary Surgery
			Skin	Corpus/Penile Shaft			
**5 y**	Exprophy vesica, aphallia	No cavernous tissue	Local scrotal skin flap	Scrotal flap phalloplasty	Incontinent	Poor	Bladder neck closure
**17 y**	Trauma	Total penile amputation, no cavernous tissue	Forearm free skin flap	Microsurgical free radial forearm flap phalloplasty	Incontinent	Poor	Urethral stricture, urethroplasty, vesicostomy
**3 y**	Monopolar cautery burn	Partial penile amputation	Local skin graft	Cavernous body dissection,	Continent	Adequate	-
**6 m**	Monopolar cautery burn	Total penile amputation, no cavernous tissue	Local skin graft	Cavernous body dissection	Continent	Adequate	-
**5 y**	Hypospadias surgery	Partial glans loss, penile skin loss	Local skin graft	Glans and penile skin reconstruction	Continent	Good	-
**8 y**	Fournier gangrene (Burkitt lymphoma)	Glans loss, total penile skin loss	Local skin graft	Glans and penile skin reconstruction	Continent	Good	-
**6 y**	Gun shot	Partial penile amputation	Local skin graft	Cavernous body dissection	Continent	Good	-
**5 y**	Extrophy cloaca	Partially existing corpus cavernousum	Local skin graft	Cavernous body dissection	Incontinent	Poor	-
**2 y**	Rudimentary penis	No cavernous tissue	Suprapubic skin flap	Local skin flap phalloplasty	Continent	Poor	Urethral dilatation
**5 y**	Extrophy vesica, diphallia	Two separate cavenous tissues	Local skin graft	Excision of non-functional cavernous tissue and reconstruction with local skin graft	Continent	Excellent	-

## Data Availability

The raw data supporting the conclusions of this article will be made available by the authors on request.
